# Myeloid Heme Oxygenase-1 Haploinsufficiency Reduces High Fat Diet-Induced Insulin Resistance by Affecting Adipose Macrophage Infiltration in Mice

**DOI:** 10.1371/journal.pone.0038626

**Published:** 2012-06-21

**Authors:** Jun-Yuan Huang, Ming-Tsai Chiang, Shaw-Fang Yet, Lee-Young Chau

**Affiliations:** 1 Graduate Institute of Life Sciences, National Defense Medical Center, Taipei, Taiwan, Republic of China; 2 Institute of Biomedical Sciences, Academia Sinica, Taipei, Taiwan, Republic of China; 3 National Health Research Institutes, Zhunan, Taiwan, Republic of China; University of Padova, Italy

## Abstract

Increased adipose tissue macrophages contribute to obesity-induced metabolic syndrome. Heme oxygenase-1 (HO-1) is a stress-inducible enzyme with potent anti-inflammatory and proangiogenic activities in macrophages. However, the role of macrophage HO-1 on obesity-induced adipose inflammation and metabolic syndrome remains unclear. Here we show that high-fat diet (HFD) feeding in C57BL/6J mice induced HO-1 expression in the visceral adipose tissue, particularly the stromal vascular fraction. When the irradiated C57BL/6J mice reconstituted with wild-type or HO-1^+/−^ bone marrow were fed with HFD for over 24 weeks, the HO-1^+/−^ chimeras were protected from HFD-induced insulin resistance and this was associated with reduced adipose macrophage infiltration and angiogenesis, suggesting that HO-1 affects myeloid cell migration toward adipose tissue during obesity. In vivo and in vitro migration assays revealed that HO-1^+/−^ macrophages exhibited an impaired migration response. Chemoattractant-induced phosphorylation of p38 and focal adhesion kinase (FAK) declined faster in HO-1^+/−^ macrophages. Further experiments demonstrated that carbon monoxide and bilirubin, the byproducts derived from heme degradation by HO-1, enhanced macrophage migration by increasing phosphorylation of p38 and FAK, respectively. These data disclose a novel role of hematopoietic cell HO-1 in promoting adipose macrophage infiltration and the development of insulin resistance during obesity.

## Introduction

Obesity is a major cause for insulin resistance, which is a key risk factor of metabolic syndrome [1]. Compelling evidence has revealed that obesity is associated with a chronic state of oxidative stress and low-grade inflammation, which is accompanied by increased macrophage infiltration in adipose tissues [2], [3]. The dysregulated expression of adipokines and inflammatory cytokines by adipocytes and infiltrating macrophages in adipose tissue contributes to obesity-induced insulin resistance [2], [3]. It has been shown that the infiltrating macrophages in obese adipose tissue are derived from circulating monocytes [4]. Several chemotactic factors, including monocytic chemotactic protein-1 (MCP-1), osteopontin, CXC motif chemokine ligand-14, and angiopoietin-like protein 2, are upregulated in adipose tissue during the early phase of obesity and mediate the recruitment of monocytes to adipose tissue, and genetic ablation of these genes ameliorates obesity-induced adipose inflammation and insulin resistance in animals [5], [6], [7], [8]. These findings highlight the significance of adipose macrophage infiltration in the development of obesity-associated metabolic syndrome.

Recently, studies have shown that the resident macrophages in lean adipose tissue express genes with characteristics of M2 or alternatively activated macrophages [9], [10], [11]. Obesity induces a phenotypic switch of the adipose macrophages from M2 state to the M1 or classical activated macrophages expressing CD11c and proinflammatory cytokines [9], [10], [11]. Depletion of CD11c-positve cells has been shown to normalize the insulin sensitivity in obese mice [12]. Whereas, disruption of PPARgamma or PPARdelta gene in macrophages impairs M2 macrophage activation and predisposes the animals to develop insulin resistance [13], [14], [15], [16]. These findings support the pathophysiological significance of adipose macrophage polarization in the regulation of insulin resistance.

In addition to macrophage infiltration, adipose tissue expansion during obesity is accompanied with vascular remodeling [17], [18]. Treatment of mice with angiogenesis inhibitor prevents the increase of white adipose mass induced by high fat diet (HFD) and in genetic mouse model, and increases insulin sensitivity of HFD-fed mice [19], [20]. These observations indicate that angiogenesis is crucial for the development of obesity and associated insulin resistance. It has been shown that the bone marrow-derived LYVE-1(+) macrophages recruited to the tip region of adipose tissue have an angiogenic role in the outgrowth of adipose tissue under normal circumstances [21]. As activated macrophages can produced multiple angiogenic factors [17], [22], it is conceivable that HFD-induced increase of adipose macrophages may influence the development of obesity via influencing adipose angiogenesis.

Heme oxygenase-1 (HO-1) is a stress-inducible enzyme catalyzing the oxidative degradation of heme to release free iron, carbon monoxide (CO), and biliverdin [23]. In addition to its role in heme catabolism, HO-1 plays important roles in various pathophysiological states associated with cellular stress. Numerous studies have demonstrated that biliverdin and its metabolite, bilirubin, are antioxidants, and CO exerts a potent anti-inflammatory effect in various disease settings [23]. Systemic induction of HO-1 by treatment with hemin or cobalt protoporphyrin in ob/ob mice or Zucker rats has been shown to reduce adiposity and improve insulin sensitivity in the diabetic animals [24], [25], [26], suggesting the potential of HO-1 as a therapeutics for type II diabetes. The protective effect of systemic HO-1 was shown to be mediated by an increase in adiponectin expression, enhanced AMP kinase activation in both adipocytes and skeletal muscles, and suppression of adipogenesis and inflammatory cytokine expression. Whether systemic HO-1 has an impact on adipose macrophages in these experimental settings is not clear. A HO-1 null mutation in macrophages has been shown to be associated with increased levels of reactive oxygen species and inflammatory cytokines following stimulation by oxidized low-density lipoprotein [27]. HO-1 induction is implicated in the polarization of macrophages toward the anti-inflammatory M2 phenotype induced by apoptotic cells [28]. Nevertheless, the effect of HO-1 on adipose macrophage polarization and associated inflammation during obesity has not yet been explored. On the other hand, HO-1 induces vascular endothelial growth factor (VEGF) in macrophages and promotes angiogenesis during wound healing and tissue remodeling [29]. In this regard, macrophage HO-1 may influence obesity through increasing adipose angiogenesis. In order to explore the pathophysiological role of macrophage HO-1 in this disease setting, we performed a bone marrow transplantation (BMT) experiment on irradiated B6 mice with bone marrow cells isolated from wild-type (WT) or HO-1^+/−^ mice to assess the role of hematopoietic HO-1 on HFD-induced adipose inflammation and insulin resistance in animals. Our data showed that hematopoietic HO-1 haploinsufficiency protected mice from obesity-induced insulin resistance by reducing adipose macrophage infiltration and inflammation. Additional experiments were also performed to reveal the underlying mechanism involved.

## Results

### HFD Induces HO-1 Expression in White Adipose Tissue

**Figure 1:**
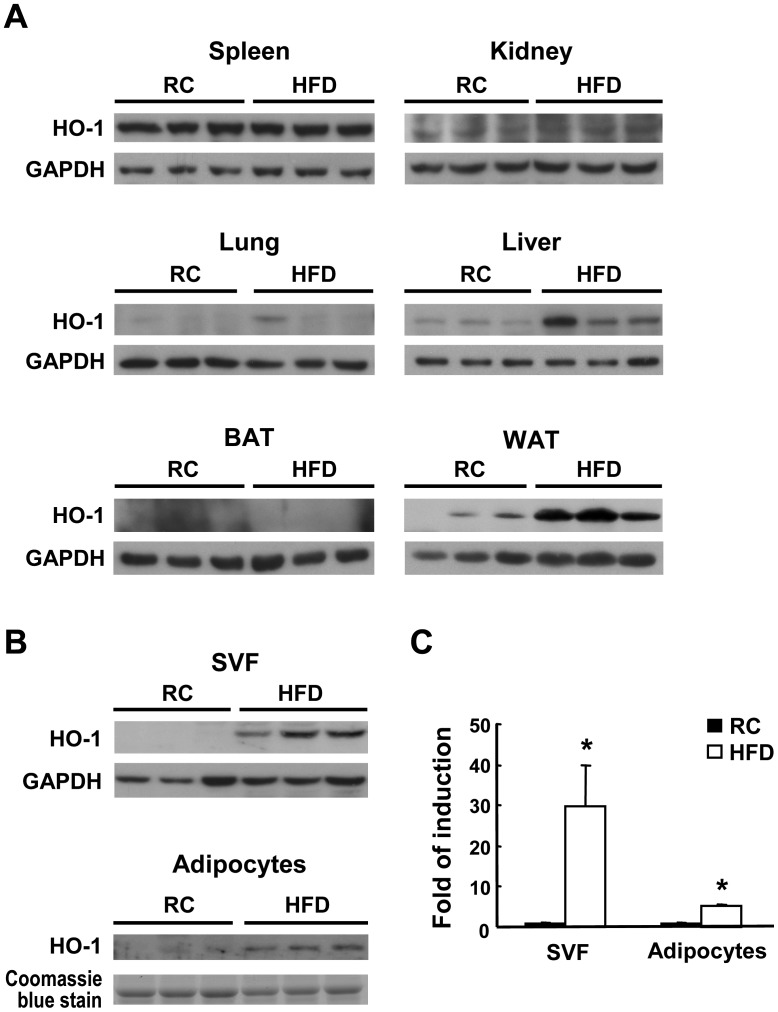
Effect of HFD feeding on HO-1 expression in various tissues. Male B6 mice (8-week-old) were fed a regular chow (RC) diet or a HFD for 12 weeks, then were sacrificed. A, HO-1 protein expression in the spleen, kidney, lung, liver, brown fat tissue (BAT), and white fat tissue (WAT) of three mice per group examined by Western blot analysis. B, The SVF and adipocytes from the visceral fat tissue were isolated from three individual mice of each group and subjected to Western blot analysis. C, HO-1 levels were quantified by densitometry. *P<0.05 vs the chow diet-fed group.

To examine whether HO-1 expression is induced by HFD, we performed Western blot analysis on various tissues isolated from C57BL/6J mice fed a regular chow diet or a HFD for 3 months. As shown in Fig. 1A, HO-1 expression in the spleen, lung, kidney, and brown adipose tissue was not significantly affected by HFD and only moderately increased in the liver. However, HO-1 expression in visceral white adipose tissue (WAT) was significantly higher in the HFD-fed group. Further experiment revealed that HFD-induced HO-1 expression was substantially higher in the stromal vascular fraction (SVF) than in the adipocyte fraction isolated from the WAT of obese mice (Fig. 1B & 1C).

### Hematopoietic HO-1 Haploinsufficiency Protects Mice from HFD-induced Insulin Resistance

**Figure 2:**
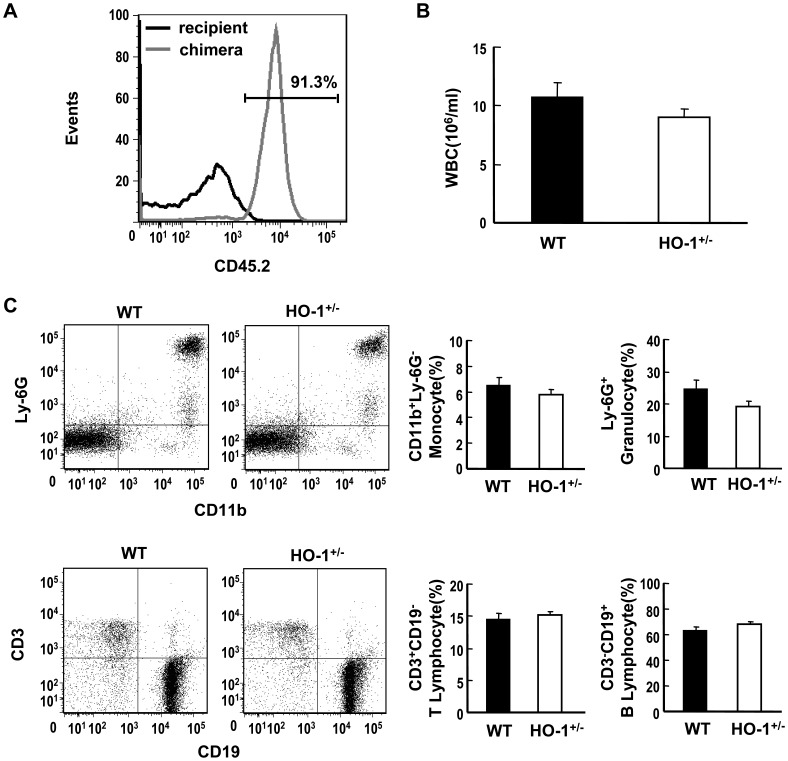
Generation of hematopoietic HO-1^+/−^ chimeric mice. A, C57BL/6 mice (B6.SJL-*Ptprc^a^ Pepc^b^*/BoyJ ) expressing CD45.1 alloantigen (Ly5.1) on the surface of circulating leukocytes were lethally irradiated, followed by transplantation of bone marrow cells isolated from C57BL/6J mice expressing CD45.2. After 4 weeks, whole blood were collected from chimeric mice and analyzed for the expression of CD45.2 by flow cytometry. The blood cells isolated from un-irradiated recipient mice were used as control. Data shown is a representative of 3 mice with independent BMT. B, C57BL/6J mice were lethally irradiated and received transplantation of bone marrow cells isolated from WT and HO-1^+/−^ mice. At 4 weeks after BMT, peripheral blood was collected and white blood cell count was determined. The number of mice in each chimeric group is five. C, The percentages of various blood cell types were analyzed by flow cytometry. The number of mice analyzed in each group is five.

**Figure 3:**
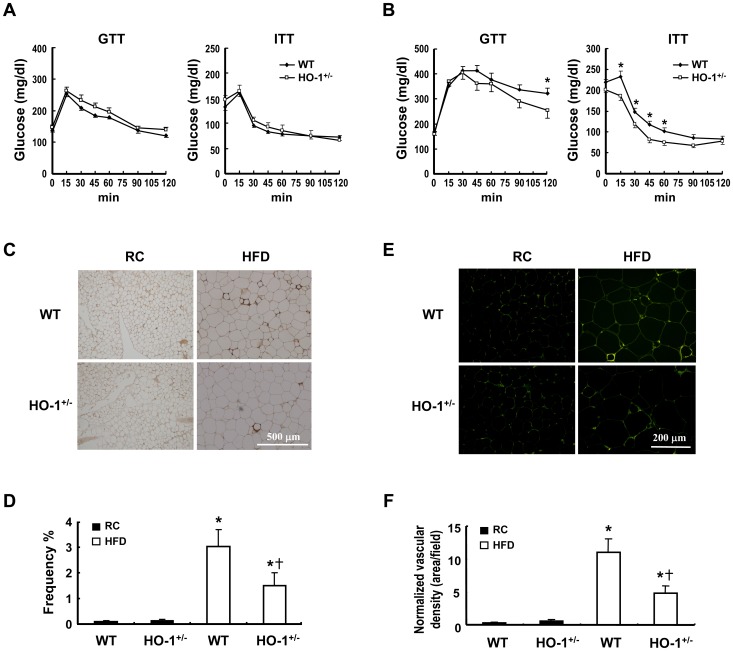
HO-1 haploinsufficiency in hematopoietic cells protects mice from HFD-induced insulin resistance. A and B, Chimeric mice with transplanted WT or HO-1^+/−^ bone marrow were fed a regular chow (RC) diet (A) or a HFD (B), then a glucose tolerance test (GTT) and an insulin tolerance test (ITT) were performed at week 28. The number of animals in each group was nine or ten. *P<0.05 vs WT chimeras. C and E, The animals were sacrificed after 36 weeks on the different diets and the visceral fat tissue immunostained using antibody against F4/80 (C) or CD31(E) as described in the Materials and Methods. D, F4/80-positive macrophages were quantified as the percentage of adipocytes surrounded by a crown-like structure in the total adipocytes. F, Normalized vascular density was quantified as the CD31-positive area divided by adipocyte density per field. *P<0.05 vs the chow diet-fed group of the same genotype; ^†^ *P*<0.05 vs HFD-fed WT group.

Bone marrow-derived macrophages represent a significant proportion of the cells recruited to the SVF of adipose tissue during obesity [4]. To evaluate the effect of HO-1 in hematopoietic cells on adipose macrophage infiltration and insulin resistance in HFD-fed mice, we performed BMT experiments to generate chimeric mice bearing wild-type (WT) or HO-1^+/−^ bone marrow. As the B6 strain mouse is the most commonly used model for HFD-induced obesity, the HO-1^+/−^ mice, originally on the B6/129SV mixed genetic background, were backcrossed six generations onto the C57BL/6J genetic background prior to be used as donors for the BMT to C57BL/6J recipient mice. The reason for using bone marrow from HO-1^+/−^ but not HO-1^−/−^ mice for experiments is two-fold. First, HO-1 deficiency causes partial embryonic lethality [30], which is exacerbated in C57BL/6J genetic background. Consistent with previous report by others [31], we had not been able to obtain any viable HO-1^−/−^ pup from the HO-1^+/−^×HO^+/−^ breeding pairs over one year of time, which precluded us from conducting the experiments with HO-1^−/−^ mice. Second, HO-1 deficiency is not very common in humans. Although the impact of HO-1 haploinsufficiency is less profound, study with heterozygote knockout mice would be more relevant to human situation. To confirm the reconstitution efficiency in chimeras, we performed BMT using congenic donors and recipients expressing different lymphocyte surface markers CD45.2 and CD45.1, respectively. Flow cytometric analysis of the circulating leukocytes isolated from the chimeric mice at 4 weeks after BMT revealed over 90% reconstitution (Fig. 2A). To examine whether hematopoietic HO-1 haploinsufficiency has an effect on leukocyte differentiation, the percentages of different cell types in the circulating bloods of WT and HO-1^+/−^ chimeras were also analyzed. As shown in Fig. 2B & 2C, there was no significant difference in the total white cell count and the percentages of lymphocytes, granulocytes, and monocytes between WT and HO-1^+/−^ chimeric mice examined at 4 weeks post BMT. The relative abundance of the circulating monocyte subsets, Ly-6C^ hi^ vs LY-6C ^low^, were also analyzed. As demonstrated in Fig. S1, no significant difference was observed between WT and HO-1^+/−^ chimeras. These results indicate that the differentiation of various blood cell types and their releases from bone marrow were not altered in HO-1^+/−^ chimeras. To examine the effect of hematopoietic HO-1 haploinsufficiency on HFD-induced insulin resistance, the chimeras were fed a regular chow diet or a HFD starting 4 weeks after BMT. Blood cell genotyping performed at 24 weeks after BMT confirmed that the reconstitution of the donor bone marrow in the recipient B6 mice maintained at least for 6 months (Fig. S2). As shown in Table 1, HFD feeding for 24 weeks resulted in a significant increase in body weight in both chimeras. The food intake and metabolic rate were similar between WT and HO-1^+/−^chimeras placed on either chow diet or HFD. However, the WT chimeras gained ∼12% more weight than the HO-1^+/−^chimeras after HFD feeding. A micro-CT scan revealed that the WAT volume was greater in HFD-fed groups than the chow diet-fed groups and was greater in the HFD-fed WT chimeras than in the HFD-fed HO-1^+/−^chimeras, indicating that the greater fat mass contributes to the greater weight gain in WT chimeras. Likewise, fasting insulin level was significantly increased in the WT chimeras after HFD feeding, but hyperinsulinemia was less evident in the HFD-fed HO-1^+/−^chimeras. The glucose tolerance test (GTT) and the insulin tolerance test (ITT) revealed no significant difference between the chow diet-fed WT and HO-1^+/−^chimeras (Fig. 3A). Whereas, when the HFD-fed chimeras were tested, the WT chimeras showed much slower glucose clearance and were less responsive to insulin (Fig. 3B).

**Table 1:** Metabolic parameters of the WT and HO-1^+/−^ chimeras fed regular chow diet or HFD.

Diet	Regular chow		HFD	
BM genotype	WT (n = 9)	HO-1^+/−^ (n = 10)	WT (n = 10)	HO-1^+/−^(n = 10)
Body weight (g)	25.10±0.69	25.57±0.44	34.00±1.54#	30.48±1.36^#^ §
Food intake (g/d/mouse)	2.89±0.11	3.08±0.08	2.37±0.04#	2.11±0.03#
MR (kcal/h/kg)	16.18±0.7	16.2±0.1	13.68±0.6#	13.32±0.7#
WAT (mm^3^)	1601±277	1620±237	11974±1123#	8826±1386^#^ §
Serum cholesterol (mg/dl)	107.3±6.7	88.7±3.3	193.9±7.4#	192.0±6.5#
Serum triglyceride (mg/dl)	58.9±2.3	57.7±2.4	61.7±2.7	50.9±2§
Serum glucose (mg/dl)	133.1±7.66	147.6±8.68	165.0±3.8#	159.9±5.54
Serum insulin (ng/ml)	0.58±0.16	0.69±0.16	1.30±0.18#	0.84±0.12§

The number of animals in each group is indicated in parenthesis. The data are expressed as the mean ± SEM.

# Significant difference between different diets within the same genotype.

§ Significant difference between different genotypes on the same diet. p<0.05. Body weight, metabolic rate (MR), and WAT volume were measured after 24 weeks on the diets, while serum was collected at 26 weeks.

### Hematopoietic HO-1 Affects Macrophage Infiltration and Inflammatory Gene Expression in Obese Adipose Tissue

**Figure 4:**
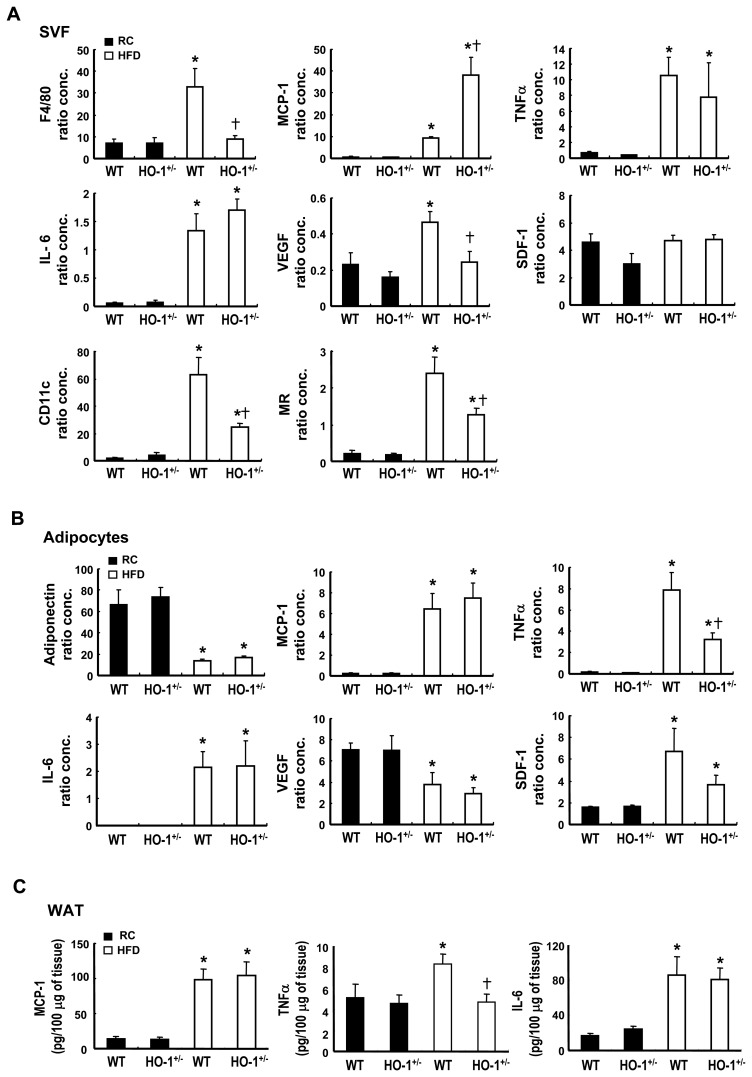
Inflammatory gene expression profiles of SVF and adipocytes from WT and HO-1^+/−^chimeras. Chimeric mice transplanted with WT or HO-1^+/−^ bone marrow were fed a regular chow (RC) diet or a HFD for 36 weeks, then the animals were sacrificed, the visceral adipose tissues collected, and the SVF and adipocytes isolated as described in Materials s and Methods. The expressions of the indicated genes in the SVF (A) and adipocytes (B) were then quantified by real time RT-PCR. The expression level of 36B4 gene was used as an internal control for normalization. The number of animals in each group was four to seven. *P*<0.05 vs the chow diet-fed group of the same genotype; ^†^ *P*<0.05 vs the HFD-fed WT group. C, The protein levels of proinflammatory cytokines in extracts of adipose tissues were determined by ELISA as described in Materials and Methods. *P*<0.05 vs the chow diet-fed group of the same genotype; ^†^ *P*<0.05 vs the HFD-fed WT group.

Pathological assessment of the visceral WAT of these chimeras after 36 weeks of HFD feeding revealed substantial enlargement of the adipocytes, but no significant difference between the WT and HO-1^+/−^chimeras (Fig. S3A). Immunostaining experiments performed with antibody against the macrophage surface marker, F4/80, showed that the extent of macrophage infiltration, characterized by a crown-like structure surrounding dead adipocytes, was significantly higher in the WAT of the HFD-fed WT chimeras than in the corresponding HO-1^+/−^chimeras (Fig. 3C & 3D). We also performed immunostaining with antibody against CD31, a specific marker of endothelial cells. As shown in Fig. 3E & 3F, the vascular density, after normalization by the adipocyte density (Fig. S3B & S3C), in the adipose tissue was significantly increased in HFD-fed chimeras and was much higher in the HFD-fed WT chimeras than in the HFD-fed HO-1^+/−^chimeras. To assess the effect of the infiltrating WT and HO-1^+/−^ macrophages on adipose inflammation, we performed quantitative RT-PCR to analyze inflammatory gene expression in the SVF and adipocyte fractions of these mice. The results showed that, in the SVF, the HFD induced high expression of inflammatory cytokine genes, including tumor necrosis factor-α (TNF-α), interleukin-6 (IL-6), and MCP-1 (Fig. 4A). However, when TNF-α and IL-6 mRNA levels in the HFD-fed chimeras were compared, there was no significant difference between these two groups of mice, but, interestingly, MCP-1 expression was significantly higher in the SVF of the HFD-fed HO-1^+/−^chimeras. In parallel with the higher degree of macrophage infiltration and vascularization in the WAT of the HFD-fed WT chimeras, levels of F4/80 and VEGF mRNAs in the SVF of this group of mice were substantially higher than those in the corresponding HO-1^+/−^chimeras, whereas chemokine stromal cell-derived factor (SDF-1) mRNA levels showed no substantial difference (Fig. 4A). We also examined the levels of CD11c and mannose receptor, which are the surface markers for M1 and M2 macrophages, respectively. In parallel with the expression level of F4/80, both expression levels of CD11c and mannose receptors were markedly increased in the SVF of the HFD-fed chimeras. Moreover, both expression levels were significantly higher in the SVF of the HFD-fed WT chimeras as compared to those in the corresponding HO-1^+/−^counterparts (Fig. 4A). In the adipocyte fraction, gene expression analysis revealed that the HFD significantly increased levels of mRNAs coding for TNF-α, IL-6, MCP-1, and SDF-1. The increase in TNF-α mRNA was much greater in the WT chimeras than the HO-1^+/−^ chimeras, whereas IL-6, MCP-1, and SDF-1 mRNA levels showed no significant difference between these two groups (Fig. 4B). In contrast, adiponectin and VEGF mRNA levels were significantly decreased by the HFD, with no significant difference between the two chimeras (Fig. 4B). Consistent with the mRNA expression levels in adipocytes, the protein levels of TNF-α, IL-6 and MCP-1 determined by ELISA were substantially higher in the WAT of HFD-fed chimeras as compared to these of chow diet-fed counterparts (Fig. 4C). Moreover, HFD-induced increase of TNF-α protein expression was significantly greater in WT chimeras than in HO-1^+/−^ chimeras.

### Hematopoietic HO-1 has No Effect on HFD-induced Liver Steatosis

**Figure 5:**
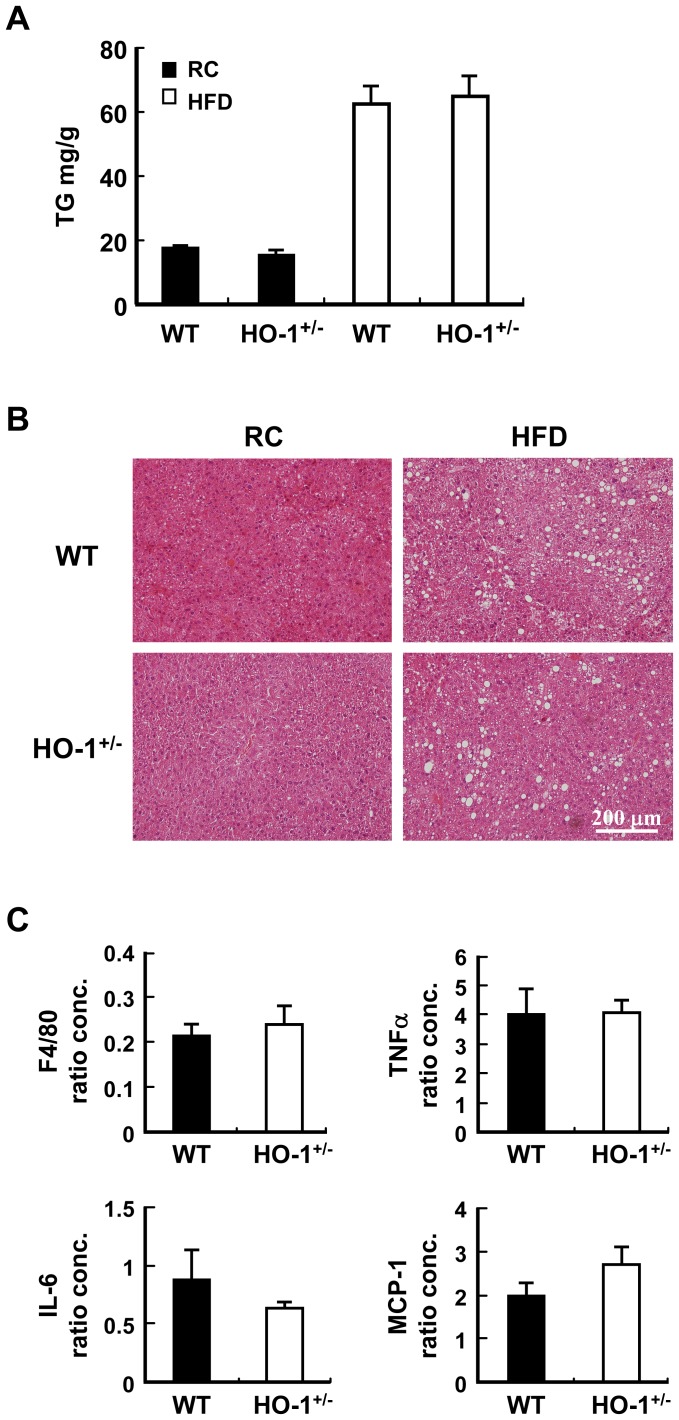
Hematopoietic HO-1 haploinsufficiency has no effect on HFD-induced liver steatosis. Chimeric mice harboring WT or HO-1^+/−^ bone marrow were subjected to chow diet or HFD for 36 weeks. A, Liver tissues were collected and the TG contents were determined. The number of mice in each group is nine to ten. B, The representative images of the H & E stain. C, The expression levels of indicated genes in the livers of HFD-fed WT and HO-1^+/−^ chimeras. The number of animals in each group is five.

To assess whether hematopoietic HO-1 has an impact on liver steatosis in obese mice, the liver triglyceride (TG) level was determined. As shown in Fig. 5A, liver TG was significantly increased by HFD. Nevertheless, hematopoietic HO-1 haploinsufficiency did not affect the increase of liver TG in HFD-fed chimeras. This was further confirmed by the H&E staining, which showed similar severity of liver steatosis in both HFD-fed chimeras (Fig. 5B). Likewise, analysis of hepatic inflammatory gene expressions did not reveal significant difference in the expressions of F4/80, TNF-α, IL-6. and MCP-1 between HFD-fed WT and HO-1^+/−^ chimeras (Fig. 5C).

### HO-1 Affects Macrophage Migration Response in vivo and in vitro

**Figure 6:**
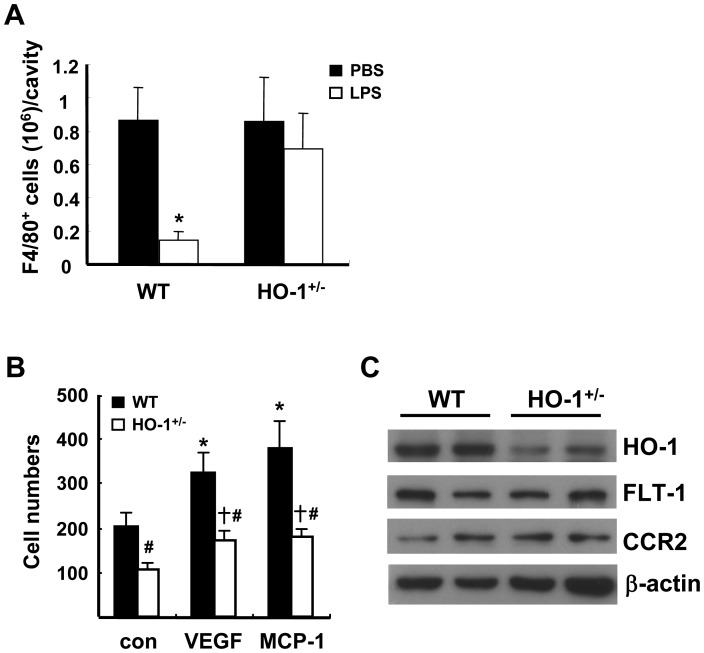
Hematopoietic HO-1 haploinsufficiency impairs macrophage migration. A, Thioglycollate-induced peritoneal macrophages from WT and HO-1^+/−^ mice were isolated and quantified 4 h after the mice received an intraperitoneal injection of PBS or LPS (1.25 µg). The number of animals was six for each group of the WT mice and nine for each group of the HO-1^+/−^ mice. *P<0.05 vs the PBS-treated group of the same genotype. B, Peritoneal macrophages isolated from WT and HO-1^+/−^ mice were tested in an in vitro transwell migration assay. The macrophages isolated from each individual mouse were tested for their migration response to 10 ng/ml of MCP-1 or VEGF as indicated; Con are controls. The number of animals in each group was six. *P<0.05 vs the WT control macrophages; ^†^ *P*<0.05 vs the HO-1^+/−^ control macrophages; ^#^ *P*<0.05 vs WT macrophages receiving the same treatment. C, HO-1, CCR2, and Flt-1 protein levels in WT and HO-1^+/−^ macrophages examined by Western blot analysis.

To explore the mechanism underlying the reduced adipose macrophage infiltration in the HFD-fed HO-1^+/−^ chimeras, we performed an in vivo migration assay in WT and HO-1^+/−^ mice by measuring LPS-induced efflux of thioglycollate-induced macrophages from the peritoneal cavity [32]. As shown in Fig. 6A, the numbers of F4/80-positve macrophages accumulated in the peritoneal cavities at 4 days after thioglycollate injection were comparable in WT and HO-1^+/−^ mice. Lipopolysaccharide (LPS) treatment for 4 hr led to a substantial reduction of macrophages in the peritoneal cavity in WT mice, whereas no significant LPS-induced macrophage efflux was observed in HO-1^+/−^ mice. We next performed an in vitro migration assay using peritoneal macrophages isolated from WT and HO-1^+/−^ mice. As shown in Fig. S4A, HO activity was significantly lower in HO-1^+/−^ macrophages as compared to that of WT counterparts. Moreover, the level of reactive oxygen species (ROS) detected by a redox-sensitve fluorescent dye was significantly higher in HO-1^+/−^ macrophages (Fig. S4B & S4C), indicating that HO-1 haploinsufficiency has an impact on the redox state of macrophages. When the migration responses of WT and HO-1^+/−^ macrophages following VEGF or MCP-1 stimulation were examined using the transwell migration system, the results showed that WT macrophages exhibited better basal migration than HO-1^+/−^ macrophages and also showed better VEGF or MCP-1-induced migration (Fig. 6B). We also performed the migration assay using the monocytes isolated from circulating blood. As demonstrated in Fig. S5A, the CD11b^+^ Ly-6G^−^ monocytes isolated from WT and HO-1^+/−^ mice were over 90% purity. Similar to peritoneal macrophages, HO-1^+/−^ monocytes exhibited significantly lower migration response to MCP-1 as compared to WT monocytes (Fig. S5B). Western blot analysis demonstrated that levels of the MCP-1 receptor CCR2 or the VEGF receptor-1 Flt-1 were comparable in the WT and HO-1^+/−^ macrophages (Fig. 6C). These results indicate that HO-1 haploinsufficiency impaired the migration ability of monocytes/macrophages.

### HO-1 Modulates the Signaling Pathways Implicated in the Migration Response

**Figure 7:**
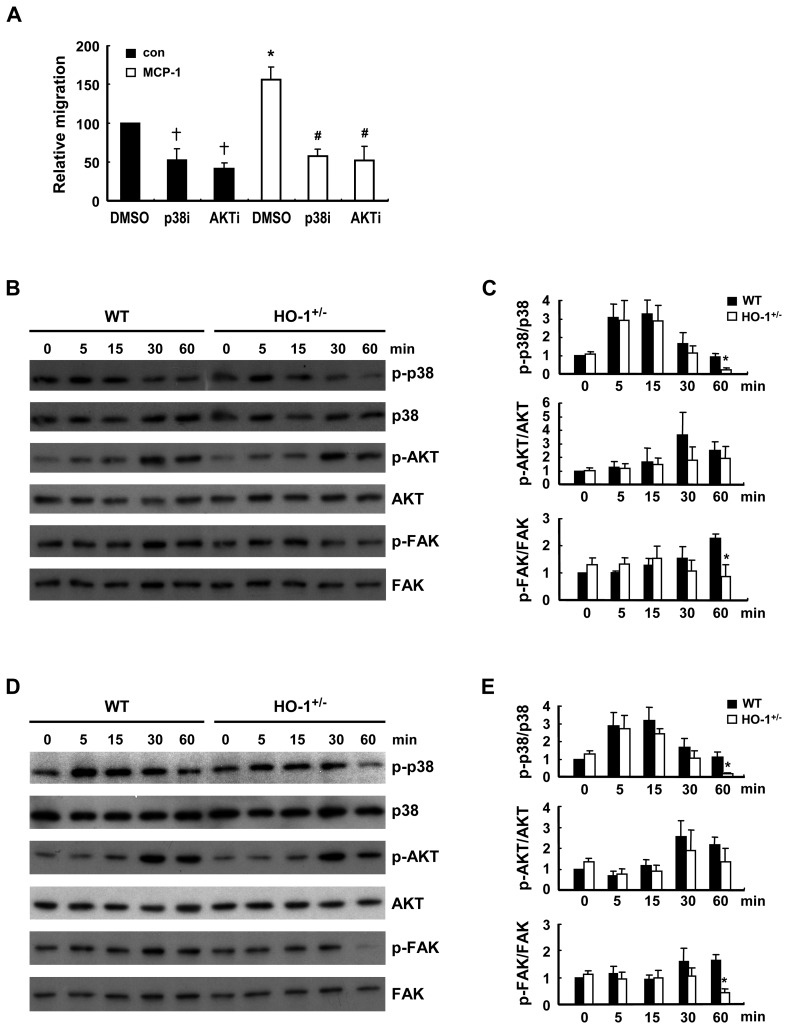
HO-1 influences chemoattractant-induced signaling. A, WT peritoneal macrophages were tested in an MCP-1 (10 ng/ml)-induced transwell migration assay in the absence or presence of SB203580 (20 µM; p38i)) or Akt inhibitor VIII (10 µM; AKTi). The data are the mean ± SEM for three independent experiments. * and ^†^ *P*<0.05 vs the corresponding control group; ^#^ *P*<0.05 vs the MCP-treated group. B-E, Peritoneal macrophages isolated from WT and HO-1^+/−^ mice were stimulated with 10 ng/ml of MCP-1 (B &C) or VEGF (D &E) for the indicated times, then the phosphorylation status of the indicated kinases was examined by Western blot analysis (B & D) and levels of phospho-p38, phospho-AKt, and phospho-FAK quantified by densitometry (C & E). The data were presented as the normalized intensity relative to that of the zero-time point WT control group, which was set to 1. The results shown are the mean ± SEM for three to four independent experiments. * *P*<0.05 vs WT macrophages.

Cell migration is a complex process regulated by the activation of various kinase signaling pathways. As HO-1 has been shown to modulate p38 and AKT signaling in various cellular contexts [23], we first examined whether the basal and MCP-1-induced migration responses of macrophages from WT mice were affected by the inhibition of p38 or/and AKT activation. As shown in Fig. 7A, both basal and MCP-1-induced migration was substantially inhibited by SB203580 and Akt inhibitor VIII, the pharmacological inhibitors of p38 and AKT activation, respectively. To assess whether the effect of HO-1 on macrophage migration was mediated through p38 and AKT-dependent signaling, MCP-1-induced phosphorylation of p38 and AKT was compared in WT and HO-1^+/−^ macrophages. The results showed that MCP-1 induced phosphorylation of p38 and AKT, which was first evident at 5 and 30 min, respectively, then gradually decayed (Fig. 7B & 7C). After 60 min of MCP-1 stimulation, phosphorylated p38 level in HO-1^+/−^ macrophages was much lower than that in WT macrophages, indicating that phosphorylated p38 decayed more rapidly in HO-1^+/−^ macrophages. Whereas, AKT phosphorylation levels at 30 min and 60 min of MCP-1 stimulation were not significantly different between WT and HO-1^+/−^ macrophages. The effect of HO-1 on FAK tyrosine phosphorylation in macrophages, one of the key signaling events leading to actin polymerization [33], [34], was also examined. As shown in Fig. 7B and 7C, FAK phosphorylation was significantly increased at 30 min and sustained for up to at least 60 min following MCP-1 treatment in WT macrophages, but no significant increase was seen in HO-1^+/−^ macrophages. When WT and HO-1^+/−^ macrophages were subjected to VEGF treatment, similar results were obtained (Fig. 7D & 7E).

### CO and Bilirubin Promote Macrophage Migration by Modulating p38 and FAK Phosphorylation

**Figure 8:**
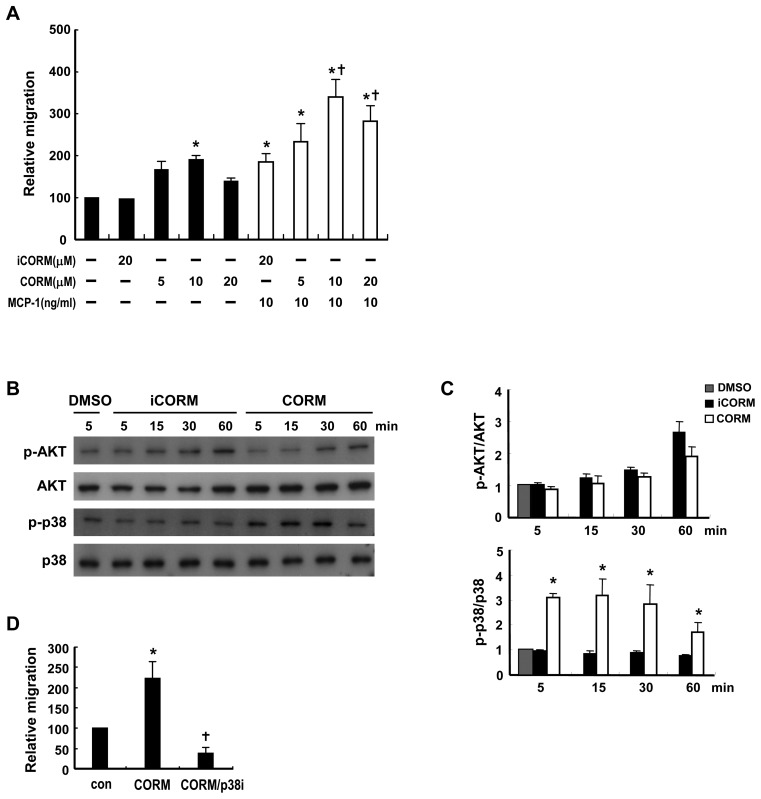
CO promotes macrophage migration by modulating p38 signaling. A, Peritoneal macrophages from WT mice were tested in a transwell migration assay without or with MCP-1 (10 ng/ml). Macrophages in the upper chamber were concurrently treated without or with the indicated concentrations of CORM-2 or iCORM-2. The data are the mean ± SEM for three independent experiments. * *P*<0.05 vs the untreated control.; ^†^ *P*<0.05 vs the MCP-1-treated cells. B, WT macrophages were treated with 10 µM iCORM-2 or CORM-2 for the indicated time, then phosphorylation of p38 or AKT was examined by Western blot analysis. C, The levels of phospho-p38 and phospho-AKT were quantified by densitometry. The data were presented as the normalized intensity relative to that of DMSO-treated group, which was set to 1. The results shown are the mean ± SEM for three independent experiments. * *P*<0.05 vs iCORM-2-trerated cells. D, Transwell migration assay performed using macrophages treated without or with 10 µM CORM-2 in the absence or presence of SB203580 (20 µM). The data shown are the mean ± SEM for four independent experiments. * *P*<0.05 vs the untreated control. ^†^ *P*<0.05 vs CORM-2 treatment. The relative migration is the migration of the treated group expressed as a percentage of that of the control group.

**Figure 9:**
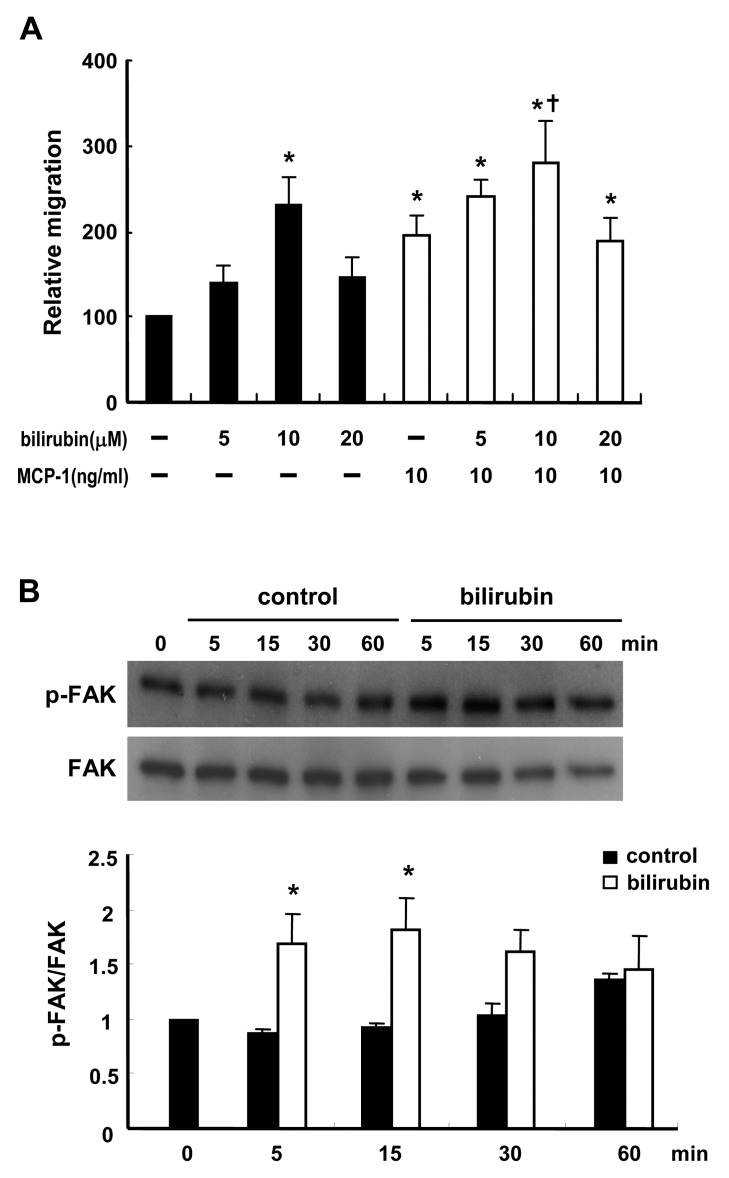
Bilirubin promotes macrophage migration by enhancing FAK phosphorylation. A, WT peritoneal macrophages were tested in a transwell migration assay without or with MCP-1 (10 ng/ml) and the macrophages in the upper chamber were concurrently treated without or with bilirubin at the indicated concentration. The data are the mean ± SEM for four independent experiments. * *P*<0.05 vs the untreated control. ^†^ *P*<0.05 vs MCP-1 treatment. B, WT macrophages were treated without or with 10 µM bilirubin for the indicated time, then phosphorylation of FAK was examined by Western blot analysis. C, The levels of phsopho-FAK were quantified by densitometry. The data were presented as the normalized intensity relative to that of the zero-time point control group, which was set to 1. The results shown are the mean ± SEM for three independent experiments. * *P*<0.05 vs the untreated control.

CO has been shown to mediate the anti-inflammatory effect of HO-1 on macrophages [35]. To explore whether CO had an effect on macrophage migration, WT macrophages were exposed to the CO-releasing compound, tricarbonyldichlororuthenium (II) dimer (CORM-2) [36]. As shown in Fig. 8A, CORM-2 promoted the basal migration response in a dose-dependent manner, with the maximal effect at 10 µM, whereas inactivated CORM-2 had no effect. MCP-1-induced migration was also enhanced by treatment of the macrophages with CORM-2. These results suggest that CO derived from heme degradation mediates, at least in part, the effect of HO-1 on macrophage migration. A further experiment demonstrated that CORM-2 treatment of macrophages induced a substantial increase in p38 phosphorylation, but not AKT phosphorylation (Fig. 8B & 8C). Treatment of macrophages with SB203580 to inhibit p38 activation also significantly suppressed CORM-2-induced macrophage migration (Fig. 8D), support a role of p38 in the CO-mediated effect. We next examined whether bilirubin, an antioxidant produced during the degradation of heme by HO-1, had an effect on macrophage migration. As shown in Fig. 9A, 10 µM bilirubin significantly increased basal migration, whereas a higher concentration (20 µM) had less effect. Treatment with 10 µM bilirubin also enhanced MCP-1-induced migration (Fig. 9A). Since ROS has been shown to downregulate FAK activation [37], we examined whether bilirubin affected FAK phosphorylation. As shown in Fig. 9B & 9C, treatment of macrophages with 10 µM bilirubin caused a transient increase in FAK phosphorylation, which was evident as early as 5 min after treatment.

## Discussion

The present study showed that HO-1 was highly induced in the WAT of HFD-fed mice. A similar observation has been reported in ob/ob mice [38]. Notably, HO-1 expression in other tissues examined was either not significantly affected or only modestly upregulated. Earlier studies by others have shown that aortic HO-1 expression was significantly reduced in ob/ob mice and Zucker fatty rats [24], [25], indicating that HO-1 is differentially regulated in different tissues in obese mice. Interestingly, HFD-induced adipose HO-1 overexpression was greatest in the SVF, which contains substantial numbers of infiltrating macrophages. Since HO-1 exerts potent anti-inflammatory and proangiogenic effects on macrophages, we were interested in exploring how its expression in hematopoietic cells affected adipose inflammation and insulin resistance during obesity. To this end, we generated chimeras reconstituted with WT or HO-1^+/−^ bone marrow and fed these mice with HFD for over 6 months. To our surprise, HFD-feeding resulted in ∼12% greater weight gain and higher adiposity in the WT chimeras than the HO-1^+/−^ chimeras even the food intakes of both chimeras were similar The metabolic rates determined in HFD-fed WT and HO-1^+/−^ chimeras also showed no significant difference. We speculate that the lean body mass, which makes a great contribution to the total energy expenditure [39], was the primary determinant of the metabolic rate in both chimeras. Nevertheless, further study is required to clarify the issue. It was found that HFD-fed WT chimeras developed hyperinsulinemia and more severe insulin resistance. Although the differences in these clinical parameters of WT and HO-1^+/−^ chimeras were considered modest, the disease manifestations in the HFD-fed mice correlated with the extent of adipose macrophage infiltration as shown immunohistochemically. The number of infiltrating macrophages was much higher in the adipose tissue of obese WT chimeras than in their HO-1^+/−^ counterparts, indicating that more WT macrophages were recruited into the adipose tissue of mice during obesity. Interestingly, the vascular density, detected by immunostaining with antibody against endothelial CD31, was also significantly greater in HFD-fed WT chimeras than HO-1^+/−^ chimeras. Since adipose tissue expansion is dependent on angiogenesis [17], the increased vascularization probably contributes to the greater adiposity seen in the HFD-fed WT chimeras. Although HO-1 has an anti-inflammatory action, we speculated that the adipose inflammation was still exacerbated by the recruitment of more macrophages in the WT chimeras than in the HO-1^+/−^ chimeras. To further assess the impacts of recruited WT and HO-1^+/−^ macrophages on the adipose inflammatory response, we analyzed inflammatory gene expression in the SVF and adipocyte fraction isolated from the adipose tissues of HFD-fed WT and HO-1^+/−^ chimeras. Correlating with the higher macrophage content of the adipose tissue in HFD-fed WT chimeras, F4/80 mRNA level was significantly higher in the SVF from this group of mice, as was VEGF mRNA level. As HO-1 has been shown to induce VEGF expression, the infiltrating WT macrophages might contribute to the higher VEGF expression in the SVF of the HFD-fed WT chimeras, which in turn enhanced the adipose angiogenesis and fat mass expansion in this group of animals. Moreover, the higher content of infiltrating WT macrophages accounted for the high levels of TNF-α and IL-6 mRNAs detected in the SVF of the HFD-fed WT chimeras, even though HO-1 is known to suppress inflammatory cytokine expression. However, MCP-1 mRNA level was much higher in the SVF from the HO-1^+/−^ chimeras. A previous study has shown that MCP-1 was robustly induced in circulating leukocytes of HO-1^−/−^ mice even in the unstressed condition [40], indicating that MCP-1 expression is most sensitive to the regulation by HO-1. Although fewer HO-1^+/−^ macrophages were recruited to the adipose tissue, it appears that the total level of MCP-1 expressed by HO-1^+/−^ macrophages still surpassed the level expressed by the high content of WT macrophages in the adipose tissue of WT chimeras. We performed additional experiments to examine whether hematopoietic HO-1 has an effect on adipose macrophage polarization during obesity. The gene expression analysis revealed that the relative expression levels of CD11c (M1) and mannose receptor (M2) were similar in the SVF fractions of HFD-fed WT and HO-1^+/−^ chimeras, indicating that WT macrophages did not exhibit a preference toward M2 polarization. Although a previous study has shown that HO-1 is implicated in apoptotic cell-induced M2 macrophage polarization [28], different mechanism may underlie macrophage polarization in the obese adipose tissue. Experiments were then performed to assess the inflammatory gene expression in the adipocyte fractions of HFD-fed chimeras. The results showed that adipocyte TNF-α mRNA level was much higher in the WT chimeras than in the HO-1^+/−^ chimeras, supporting the close link between increased macrophage infiltration and adipocyte inflammation during obesity. However, there was no significant difference in levels of mRNAs for other cytokines, such as IL-6 and MCP-1, between the WT and HO-1^+/−^ chimeras. The reason for this phenomenon is not clear. Since other immune cells are also implicated in obesity-induced adipose inflammation [41], an effect of HO-1 on other immune cells and their involvement in regulating the adipocyte inflammatory response cannot be excluded. Future study using myeloid-specific HO-1 deficient mice will be required to clarify the issue. Nevertheless,, the results from the present study clearly demonstrated that HO-1 haploinsufficiency in hematopoietic cells protected mice from HFD-induced insulin resistance, which was associated with decreased adipose macrophage infiltration and inflammation.

To further investigate the possibility that HO-1 impacts monocyte/macrophage migration to adipose tissue, we first performed the macrophage efflux assay in WT and HO-1^+/−^ mice with thioglycollate-induced peritonitis. The numbers of peritoneal macrophages examined at 4 days after thioglycollate injection in WT and HO-1^+/−^ mice were comparable. It has been shown that the extent of macrophage accumulation in the inflammatory site in the late phase is influenced by the emigration of macrophages to lymphatics [42] We speculated that HO-1 haploinsufficiency has similar effect on both migration and emigration of macrophages in the early and late phases of peritonitis, resulting in the accumulation of macrophages in peritoneum of HO-1^+/−^ mice to a similar extent as that in WT mice at 4 days after thioglycollate injection. However, a subsequent treatment of LPS induced a significant efflux of peritoneal macrophages within a short period of time in WT but not HO-1^+/−^ mice. The impaired migration response of HO-1^+/−^ macrophages was confirmed in the in vitro transwell migration assay. Similar result was observed in the migration assay with circulating monocytes isolated from WT and HO-1^+/−^ mice, indicating that HO-1 exerts same effect on the migration responses of monocytes and macrophages. Since peritoneal macrophages are easier to obtain in large quantities, we used peritoneal macrophages for the subsequent mechanistic studies. We compared cellular signaling pathways in WT and HO-1^+/−^ macrophages following stimulation with MCP-1, which induces monocyte migration via the p38- and AKT–dependent pathways [43], [44]. The results showed that MCP-1 treatment resulted in a rapid, but transient, increase in p38 phosphorylation in both WT and HO-1^+/−^ macrophages and that phosphorylated p38 levels declined more rapidly in HO-1^+/−^ macrophages. AKT phosphorylation was also induced by MCP-1, but the levels were not statistically different between WT and HO-1^+/−^ macrophages. Similar results were observed when VEGF was used as the chemoattractant. As p38-mediated signaling is implicated in chemoattractant-induced macrophage migration, we reasoned that the slower decay of p38 phosphorylation observed in WT macrophages contributed to their greater motility. Subsequent experiment demonstrated that CO, which has been shown to modulate p38 activation in macrophages [35], significantly enhanced both basal cell mobility and MCP-1-induced migration, supporting that CO mediates, at least in part, the effect of HO-1 on macrophage migration. CO has also been shown to induce the migration of endothelial progenitor cells [45]. Nevertheless, a recent study demonstrated that CO decreased platelet-derived growth factor-induced smooth muscle cell migration [46]. The effect of CO on cell motility appears to be cell type specific. We also examined the effect of bilirubin, an antioxidant produced during heme degradation by HO-1, on macrophage migration. As CO donor, bilirubin significantly enhanced macrophage migration in both the absence and the presence of MCP-1. This finding is consistent with previous reports showing that macrophage function, including motility, is significantly improved by antioxidants [47], [48]. We then showed that bilirubin treatment led to a transient increase in FAK phosphorylation, which is vital for the regulation of cell mobility. These data support the role of bilirubin in mediating part of the effect of HO-1 on macrophage migration.

In summary, the present study identified a novel function of HO-1 in macrophages. Although it is regarded as an anti-inflammatory enzyme which suppresses the production of proinflammatory cytokines in macrophages, HO-1 can impact the migration response of macrophages through modulating p38 and FAK activations. HO-1 expression in hematopoietic cells promotes the migration of macrophages toward the adipose tissue during obesity and exacerbates the development of metabolic disease. This finding is contradictory to the previous reports showing that systemic induction of HO-1 in diabetic animals reduced adiposity and improved insulin sensitivity [24], [25], [26]. It is speculated that the salutary effects of HO-1 on adipocytes and skeletal muscles as demonstrated in these earlier studies might overpower the effect of HO-1 on macrophage migration toward obese adipose tissue in their experimental settings. Moreover, the possibility that the down regulation of chemokine expression induced by HO-1 in adipocytes might restrict the adipose macrophages recruitment can not be excluded. Despite these studies supporting the protective effect of systemic HO-1 induction on obesity-induced insulin resistance, there was a report showing that the endogenous HO-derived CO was increased and promoted hypertension and endothelial dysfunction in obese Zucker rats [49]. In this regard, the regimen to increase HO-1 expression does not seem to be a superior therapeutics for metabolic diseases as suggested by previous studies [24], [25], [26]. It is apparent that future studies with cell- or tissue-specific HO-1 knockout or transgenic mice are required to delineate more precisely the differential roles of HO-1 in different cell compartments in this complicated disease.

## Materials and Methods

### Ethics Statement

All experimental procedures with animals were approved by the Institutional Animal Care and Utilization Committee of the Academia Sinica, Taiwan (Protocol #: RMiIBMCL2008070).

### Animals

HO-1^+/−^ mice, originally on the B6/129SV mixed genetic background, were backcrossed six generations onto the C57BL/6J genetic background. To generate B6 chimeric mice reconstituted with wild type (WT) or HO-1^+/−^ bone marrow, we performed BMT as described previously [50]. Briefly, bone marrow cells were obtained by blushing the femurs and tibias of WT or HO-1^+/−^ mice with PBS. Cell suspension was filtered through a 70 µm cell strainer and the mononuclear cells were isolated by Histopaque-1083 (Sigma) density gradient centrifugation. C57BL/6J recipient mice (male, 6 weeks) were lethally irradiated by 9 Gy, followed by receiving an i.v. infusion of the bone marrow-derived mononuclear cells (2×10^6^ cells/mouse) isolated from WT or HO-1^+/−^ mice. To assess the BMT efficiency, a substrain of C57BL/6 mice (B6.SJL-*Ptprc^a^ Pepc^b^*/BoyJ ) expressing CD45.1 alloantigen (Ly5.1) on the surface of circulating leukocytes were used as recipients and C57BL/6J mice expressing CD45.2 (Ly5.2) as donors. Four weeks after BMT, the circulating leukocytes were collected from the chimeric mice and the expressions of CD5.2 and CD5.1 were examined to determine the percentage of chimerism. To induce obesity in chimeric mice, at 4 weeks after BMT animals were fed either a regular chow diet or a HFD (60 kcal % D12492, Research Diets) for the indicated period of time. All mice were kept on a 12 h light-dark cycle and allowed free access to food and water. Body weight and serum glucose were measured every other week. The resting metabolic rate was measured using a metabolic respiratory system (Buxco). White adipose tissue volume was measured using the SkyScan 1076 micro-CT system. The serum insulin concentration was determined using a mouse insulin ELISA kit (Millipore). Serum cholesterol and TG concentrations were measured using a FUJI DRI-CHEM clinical chemistry analyzer. To perform the GTT and ITT, the animals were fasted for 6 h, then injected intraperitoneally with glucose (1 g/kg body weight) or insulin (0.7 U/kg), respectively.

### Isolation of Genomic DNA and Genotyping

Mouse tail snip (0.5 cm) or mouse whole blood (100 µl) was incubated with 700 µl of lysis buffer containing 50 mM Tris-HCl pH 8.0, 100 mM EDTA, 0.5% SDS and 360 µg proteinase K at 55°C for 18 h. Samples were then mixed with 700 µl of phenol/chloroform/isoamyl alcohol (25:24:1 v/v), vortexed for 3 min at room temperature and centrifuged at 15,500 xg for 10 min at 4°C. The upper layer was transferred to a new tube, mixed with equal volume of chloroform/isoamyl alcohol (24:1 v/v) and vortexed for 3 min at room temperature. Then the samples were centrifuged again, and the upper layer was mixed with equal volume of isopropanol. After gently mixing, the genomic DNA was pelleted by centrifugation at 15,500 xg for 10 min at 4°C, washed once with 75% alcohol, air dried, and resuspended in 100 µl ddH_2_O. Genotyping PCR was performed by using 50 ng genomic DNA, 62.5 pmole primer, 10 nmole dNTPs and 2.5 U FastStart Taq DNA polymerase (Roche). The primer pairs used were wild type allele (5′-GGTGACAGAAGAGGCTAAG-3′ and 5′-CTGTAACTCCACCTCCAAC-3′) and mutated allele (5′-TCTTGACGAGTTCTTCTGAG-3′ and 5′-ACGAAGTGACGCCATCTGT-3′). The PCR condition was 30 cycles of 94°C 30 sec, 58°C 30 sec, and 72°C 30 sec. The PCR product was 456 bp for wild type allele and 400 bp for mutated allele.

### HO Activity Measurement

Peritoneal macrophages were washed twice with PBS and centrifuged at 115 xg for 5 min at 4°C. The cell pellet was resuspended in 0.1 M K_3_PO_4_ pH 7.4 containing 1 mM EDTA and 0.5 mM PMSF. After sonication, the cell homogenate was centrifuged at 15,500 xg for 10 min at 4°C. The protein concentration of supernatant was determined and 1 mg of proteins was added to 0.1 M K_3_PO_4_ pH 7.4 containing 1.5 mg of rat liver cytosol, 50 µM hemin, 1 mM NADPH, 2 mM glucose-6-phosphate and 0.3 units of glucose-6-phosphate dehydrogenase. The mixture was incubated in the dark for 1 h at 37°C. Bilirubin was then extracted with 0.5 ml chloroform and quantified by the absorbance difference between 464 and 530 nm with an extinction coefficient of 40/mM*cm.

### ROS Determination

2×10^5^ peritoneal macrophages were washed twice with Hank’s Balanced Salt Solution (HBSS) and centrifuged at 115 xg for 5 min at room temperature. The cell pellet was resuspended in 0.5 ml HBSS containing 10 µM 5-(and-6)-chloromethyl-2,7-dichlorodihydrofluorescein diacetate, acetyl ester (CM-H2DCFDA) and incubated at 37°C for 1 h in dark. Cells were then washed once with HBSS and returned to prewarmed serum free RPMI 1640 medium containing 0.5% BSA for 30 min at 37°C. After incubation, macrophages were washed once with PBS/2% FBS and subjected to immunostaining with APC-conjugated anti-F4/80 antibody for 30 min at 4°C. Subsequently, cells were washed twice with PBS/2% FBS and the intensity of CM-H2DCFDA fluorescence in F4/80-positve cells was determined by BD™ LSR II flow cytometer (Becton Dickinson, San Joes, CA, USA) and data analyzed with FlowJo 7.5.5 flow cytometry analysis software.

### Isolation of Peripheral Blood Leukocytes

Peripheral blood was obtained from the tail vein and collected into EDTA-containing tubes. After a gentle mixing, the white blood cell count was determined by CELL-DYN 3700 (Abbott Park, Illinois, USA). Subsequently, whole blood was subjected to red blood cell lysis in ammonium chloride solution (BD Pharm Lyse™) for 5 min at room temperature. The cells were washed once with phosphate-buffered saline (PBS) containing 2% fetal bovine serum (FBS) and centrifuged at 300 xg for 5 min at 4°C. Cell pellet was then resuspended in PBS containing 2% FBS and analyzed by flow cytometry.

### Isolation of Peripheral Blood Monocytes

Peripheral blood was subjected to red blood cell lysis in ammonium chloride solution (BD Pharm Lyse™) for 5 min at room temperature. Cells were washed once with PBS containing 2% FBS and centrifuged at 300 xg for 5 min at 4°C. The cell pellet was resuspended in PBS containing 2% FBS and monocytes were enriched using the EasySep mouse monocyte enrichment kit (Stemcell Tech) following the manufacturer’s instructions with modifications. Briefly, 5×10^6^ white blood cells isolated from each mouse were resuspended in 50 µl of PBS/2% FBS and incubated with 2.5 µl normal rat serum and 2.5 µl monocyte enrichment cocktail in a 0.5 ml centrifuge tube at 4°C for 15 min. Cells were then washed once with PBS/2% FBS and centrifuged at 300 xg for 5 min at 4°C. The cell pellet was resuspended in 50 µl of PBS/2% FBS and incubated with 3 µl biotin selection cocktail for 15 min. Subsequently, cells were mixed with 7.5 µl of magnetic particles for 10 min and the centrifuge tube was placed into the magnet for 5 min. The enriched monocytes in the supernatant were then transferred to a new tube and ready for use.

### FACS Analysis

2×10^5^ cells were incubated with 1 µg Fc receptor block (eBioscience) in PBS/2% FBS for 15 min at 4°C, followed by incubation with 0.4 µg of fluorochrome-conjugated primary antibodies for additional 30 min at 4°C. Cells were then washed twice with PBS/2% FBS and centrifuged at 300 xg for 5 min at 4°C. Cell pellet was resuspended in PBS/2% FBS and incubated with 0.05 µg 7-Amino-Actinomycin D (7-AAD, BD Pharmingen™) for 10 min at 4°C for dead cell exclusion. Subsequently, cells were analyzed by BD™ LSR II flow cytometer (Becton Dickinson, San Joes, CA, USA) and data were acquired and analyzed using FlowJo 7.5.5 flow cytometry analysis software. Fluorescein isothiocyanate(FITC) -conjugated anti-F4/80 antibody was used for the analysis of F4/80 expression in macrophages. To determine the percentage of chimerism, peripheral blood leukocytes isolated from un-irradiated CD45.1 recipients and chimeric mice were stained with Alexa Fluor® 700-conjugated anti-CD45.2 antibody. Donor specific CD45.2 fluorescence emission was plotted with histogram. To compare the leukocyte cell populations of chimeric mice, peripheral blood leukocytes isolated from WT and HO-1^+/−^ chimeras were stained with Allophycocyanin(APC)-conjugated anti-CD11b antibody, Phycoerythrin(PE)-conjugated anti-Ly-6G antibody, APC-eFluor® 780-conjugated anti-CD3 antibody, PE-Cy7-conjugated anti-CD19 antibody and respective isotype control. Monocytes were identified as CD11b^+^Ly-6G^−^ cells Granulocytes were identified as Ly-6G^+^ cells. T lymphocytes were identified as CD3^+^CD19^−^ cells. B lymphocytes were identified as CD3^−^CD19^+^ cells. Within the monocyte population, cells were further analyzed as CD11b^+^Ly-6G^−^Ly-6C^high^ and CD11b^+^Ly-6G^−^Ly-6C^low^ subsets by APC-conjugated anti-Ly-6C antibody staining.

### Isolation of the SVF and Adipocytes

Mouse visceral adipose tissue was isolated, minced into fine pieces, and placed in Dulbecco’s Modified Eagle’s Medium containing 5% FBS. After centrifugation at 500×g for 5 min to pellet erythrocytes and other blood cells, the tissue suspension was incubated with collagenase (2 mg/ml; Sigma-Aldrich) in PBS containing 1 mM CaCl_2_, 0.5% bovine serum albumin, and 5 mM glucose at 37°C for 30 min with gentle shaking (150 rpm), then filtered through a 100 µm nylon mesh filter and centrifuged at 300 x g for 5 min at room temperature. The floating adipocytes and the pellet (SVF) were harvested, washed twice with PBS, and stored at −80°C.

### Measurement of Hepatic TG Content

Liver tissue (0.2 g) was homogenized in 4 ml chloroform/methanol (2∶1, vol/vol), followed by addition of 0.8 ml of 50 mM NaCl. Samples were vortexed and centrifuged at 720×g for 5 min. The sample (50 µl) from the organic layer was dried and re-dissolved in 20 µl of isopropanol. TG concentration was measured using TG assay kit (Cayman). All values of tissue TG content were normalized by liver weight.

### Real-time Quantitative PCR

Total RNAs were isolated using TRIzol reagent (Invitrogen). Reverse transcription was performed using 2 µg of total RNA, random primers, and Superscript III reverse transcriptase (Invitrogen). Real-time PCR was performed using a LightCycler® FastStart DNA MasterPLUS SYBR Green I kit (Roche Applied Science) on a LightCycler® Carousel-Based System (Roche Applied Science). 36B4 was used as an internal control. The primer pairs were TNFα (5′- AGACCCTCACACTCAGA-3′ and 5′- CCTTGTCCCTTGAAGAGAAC-3′), IL-6 (5′-GAGGATACCACTCCCAACAGAC C-3′ and 5′-AAGTGCATCATCGTTGTTCATACA-3′), VEGF (5′-CAGGCTGCACCCACGACAGAAG-3′ and 5′-CTATGTGCTGGCTTTGGTGAGGTTT-3′), SDF-1 (5′-ATGGACGCCAAGGTCGTCGCC-3′ and 5′-TTACTTGTTTAAAGCTTTCTC-3′), MCP-1 (5′-CTTCTGGGCCTGCTGTTCA -3′ and 5′-CCAGCCTACTCATTGGGATCA-3′), adiponectin (5′-GCAGAGATG GCACTCCTGGA-3′ and 5′-CCCTTCAGCTCCTGTCATTCC-3′), F4/80 (5′-TTTCCTCGCCTGCTTCTTC-3′ and 5′-CCCCGTCTCTGTATTCAACC -3′), and 36B4 (5′-CCCACTTACTGAAAAGG-3′ and 5′-GGCGGGATTAGTCGAA-3′). CD11c (5′-GAGAGCCCAGACGAAGACAG-3′ and 5′-CCATTTGCTTCCTCCAACAT-3′), Mannose receptor (5′-GCAAATGGAGCC GTCTGTGC-3′ and 5′-CTCGTGGATCTCCGTGACAC-3′).

### ELISA

Mouse epididymal adipose tissue was homogenized in lysis buffer containing 20 mM Tris-HCl pH 7.5, 150 mM NaCl, 1% NP-40, 1 mM CaCl_2_, 1 mM MgCl_2_, 1 mM PMSF, 10% glycerol and a protease inhibitor mixture (Calbiochem). After 10 min incubation on ice, tissue homogenates were centrifuged at 15500 xg for 10 min at 4°C. The supernatant was removed and the protein concentration was determined. One hundred µg of tissue lysates were used for the determination of TNF-α, IL-6 and MCP-1 protein contents using the indicated mouse ELISA kits (R&D Systems). Data were represented as the amount of cytokine per 100 µg tissue lysates.

### Immunohistochemistry

Adipose tissue sections (5 µm) were pretreated with 3% H_2_O_2_ for 10 min at room temperature to block endogenous peroxidase activity. After incubation with 5% normal goat serum for 30 min, sections were incubated with anti-F4/80 antibody (1∶300 dilution; Serotec) in PBS containing 1% normal goat serum for 1 h at 37°C, followed by 3 washes with PBS. Chromogenic detection of antigen was performed using an EnvisionTM Detection system (Dako) coupled with horseradish peroxidase/diaminobenzidine. The extent of macrophage infiltration was calculated as the percentage of adipocytes surrounded by a F4/80-positive crown-like structure in the total adipocytes in each field. For immunostaining of the vasculature, sections were incubated with 20 µg/ml of proteinase K (Sigma-Aldrich) at room temperature for 5 min. After a PBS wash, the sections were blocked with 5% normal goat serum, followed by incubation overnight at 4°C with antibody against CD31 (1∶100 dilution; BD Pharmingen). After 3 PBS washes, the sections were incubated with FITC-conjugated secondary antibody (1∶200 dilution; Invitrogen) at room temperature for 1 h in the dark, followed by washing, and examined by fluorescence microscopy. Vascular density was calculated as described previously [51]. Briefly, the CD31 immunofluorescence images were taken from 4 different fields per section under a fluorescence microscope (×200 magnification). MetaMorph image analysis software (Molecular Devices) was used for image analysis. The CD31^+^ area in each field was calculated as [(CD31^+^ signal – background (FITC-goat anti-rat IgG))/total pixels]×0.6183 mm^2^ (the total real area under 200× magnification). The vascular density was defined as CD31^+^ area per field (×10^−6^/um^2^). The normalized vascular density was calculated by dividing the CD31^+^ area with the total number of adipocytes per area. The adipocyte density was calculated by counting the number of adipocytes per area (×10^−6^/um^2^).

### Western Blot Analysis

Tissues and cells were homogenized and lysed in ice-cold RIPA buffer consisting of 50 mM Tris-HCl pH 7.4, 150 mM NaCl, 1% Nonidet P-40, 0.25% sodium deoxycholate, 1 mM EDTA, 1 mM PMSF, and protease and phosphatase inhibitor cocktails (Calbiochem). Tissue and cell lysates were centrifuged at 15,500×g for 15 min at 4°C, the supernatant collected, and the protein concentration determined by the Bradford method (Bio-Rad). The protein samples were then separated by electrophoresis in 10% SDS-polyacrylamide gels and transferred to Immobilon-P Membrane (Millipore). After blocking with 5% skim milk in Tris-buffered saline (TBS) containing 0.1% Tween 20 (TBST), the membranes were incubated with rabbit antibodies against HO-1 [52], CCR2 (Abcam), Flt-1 (Abcam), GAPDH (Santa Cruz), phospho-AKT (Ser473), phospho-p38 (Thr180/Tyr182), phospho-FAK (Tyr397) (Cell Signaling), and -AKT, p38, or FAK (all from Cell Signaling) in TBST containing 1% skim milk for 2 h at room temperature or 16 h at 4°C, then, after 3 washes with TBST, were incubated with horseradish peroxidase-conjugated secondary antibodies in TBST containing 5% skim milk. Bound antibody was then detected using the enhanced chemiluminescence system. For immunoblot quantification, the X-film was scanned by ScanMaker 8700 (MICROTEK, Hsinchu, Taiwan). The images were then analyzed using MetaMorph image analysis software. The band intensity of target protein was normalized by dividing its intensity value with the value of the internal control protein band in the same lane of the blot. Data were presented as the normalized intensity relative to the control group in each respective experiment.

### Mouse Peritoneal Macrophages

Mice were injected intraperitoneally with 3 ml of 3% thioglycollate After 4 days, peritoneal macrophages were isolated by washing the peritoneal cavity with RPMI 1640 medium containing 0.5% BSA. Cells were then plated on cell culture plate. After 4 h incubation, the non-adherent cells were removed by washing with culture medium and the adherent cells used as peritoneal macrophages, which were serum-deprived for 24 h prior to stimulation with various agents. For experiments using inactivated CORM-2, CORM-2 (Sigma-Aldrich) was incubated overnight at 37°C in serum-free RPMI 1640 medium containing 0.5% BSA prior to use.

### In vivo Migration Assay

The WT and HO-1^+/−^ mice received an intraperitoneal injection of 1 ml of 4% thioglycollate, then, 4 days later, were injected intraperitoneally with 250 µl of PBS or LPS (5 µg/ml). Four hours later, the peritoneal cells were harvested and the number of macrophages quantified by flow cytometry using FITC-conjugated anti-F4/80 antibody.

### In vitro Migration Assay

Cell migration was assessed using a 24-well Transwell with a pore size of 5 µm (Corning). 1×10^5^ peritoneal macrophages suspended in serum-free RPMI 1640 medium containing 0.5% BSA were added to the upper chambers and the lower chambers were filled with culture medium alone or containing the indicated chemoattractant. After 18 h incubation, cells that had migrated to the lower surface of the membrane were fixed with 4% paraformaldehyde for 10 min, stained with Giemsa for 2 h, and counted under a microscope.

### Statistical Analysis

Data are expressed as the mean ± SEM for at least three independent experiments. Student’s t test was used for statistical comparisons between two groups of data and one-way ANOVA was used for more than two groups. A *P* value <0.05 was considered statistically significant.

## Supporting Information

Figure S1 **Flow cytometric analysis of circulating monocytes from WT and HO-1^+/−^ chimeras**. C57BL/6J mice were lethally irradiated and received transplantation of bone marrow cells isolated from WT and HO-1^+/−^ mice. At 4 weeks after BMT, peripheral blood was collected and the percentages of CD11b^+^Ly-6G^−^Ly-6C^high^ and CD11b^+^Ly-6G^−^Ly-6C^low^ monocytes were analyzed by flow cytometry. The number of mice in each chimeric group is five.
(TIF)

Figure S2 **Genotype analysis of blood cells isolated from WT and HO-1^+/−^ chimeras.** C57BL/6J mice were lethally irradiated, followed by BMT with hematopoietic stem cells isolated from WT and HO-1^+/−^ mice. At 24 weeks after BMT, genomic DNA was isolated from the peripheral blood and analyzed by PCR for WT allele (456 bp) and mutant allele (400 bp).
(TIF)

Figure S3 **Effect of HFD on adipocyte hypertrophy and adipose angiogenesis in WT and HO-1^+/−^ chimeras.** Chimeric mice with transplanted WT or HO-1^+/−^ bone marrow were fed a regular chow (RC) diet or a HFD for 36 weeks. The animals were sacrificed and the visceral fat tissues were sectioned and stained with hematoxylin and eosin. A, The adipocyte size was analyzed using MetaMorph image analysis software (Molecular Devices). B, The adipocyte density was calculated by counting the number of adipocytes per area. C, Sections were immunostained with specific antibody against CD31. The CD31 immunofluorescence images were taken from 4 different fields per section under a fluorescence microscope (×200 magnification). The CD31^+^ area in each field was calculated as [(CD31^+^ signal – background (FITC-goat anti-rat IgG))/total pixels]×0.6183 mm^2^ (the total real area under 200× magnification). The vascular density was defined as CD31^+^ area per field (×10^−6^/um^2^). The number of animals in each group was nine or ten. *P<0.05 vs the chow diet-fed group of the same genotype; ^†^ *P*<0.05 vs HFD-fed WT group.
(TIF)

Figure S4 **Effect of hematopoietic HO-1 haploinsufficiency on HO activity and intracellular ROS level in macrophages.** Peritoneal macrophages were isolated from WT and HO-1^+/−^ mice receiving thioglycollate injection for 4 days. A, HO activity in whole cell lysates was determined and expressed as nmoles of bilirubin produced per mg proteins per h. The number of mice in each genotype is five. *P<0.05 vs WT group. B, The levels of ROS in WT and HO-1^+/−^ macrophages were assessed by incubation with a redox-sensitive fluorescent dye, CM-H2DCFDA, followed by flow cytometry. C, Quantitative results of CM-H2DCFDA fluorescence. The numbers of mice in WT and HO-1^+/−^ genotypes are three and four, respectively. *P<0.05 vs WT group.
(TIF)

Figure S5 **Hematopoietic HO-1 haploinsufficiency impairs monocyte migration response to MCP-1.** A, The circulating monocytes were isolated from WT and HO-1^+/−^ mice and subjected to APC-conjugated anti-CD11b and PE-conjugated anti-Ly-6G antibody staining, followed by flow cytometry. Monocytes were identified as CD11b^+^Ly-6G^−^ cells. B, WT and HO-1^+/−^ monocytes (5×10^4^) isolated from each individual mouse were tested for their migration response to 10 ng/ml of MCP-1. The experimental procedure was same as described for peritoneal macrophages except the migration time is 4 hr. The number of mice in each group is three. Data represented are the migration responses relative to the WT control group which was set to 100. *P<0.05 vs WT control; ^#^ *P*<0.05 vs WT monocytes receiving MCP-1 treatment.
(TIF)
