# Supplementary figures and images for: Myeloid Heme Oxygenase-1 Haploinsufficiency Reduces High Fat Diet-Induced Insulin Resistance by Affecting Adipose Macrophage Infiltration in Mice
Source: PLoS One. 2012 Jun 21;7(6):e38626. doi: 10.1371/journal.pone.0038626 (PMC3382977; doi:10.1371/journal.pone.0038626)

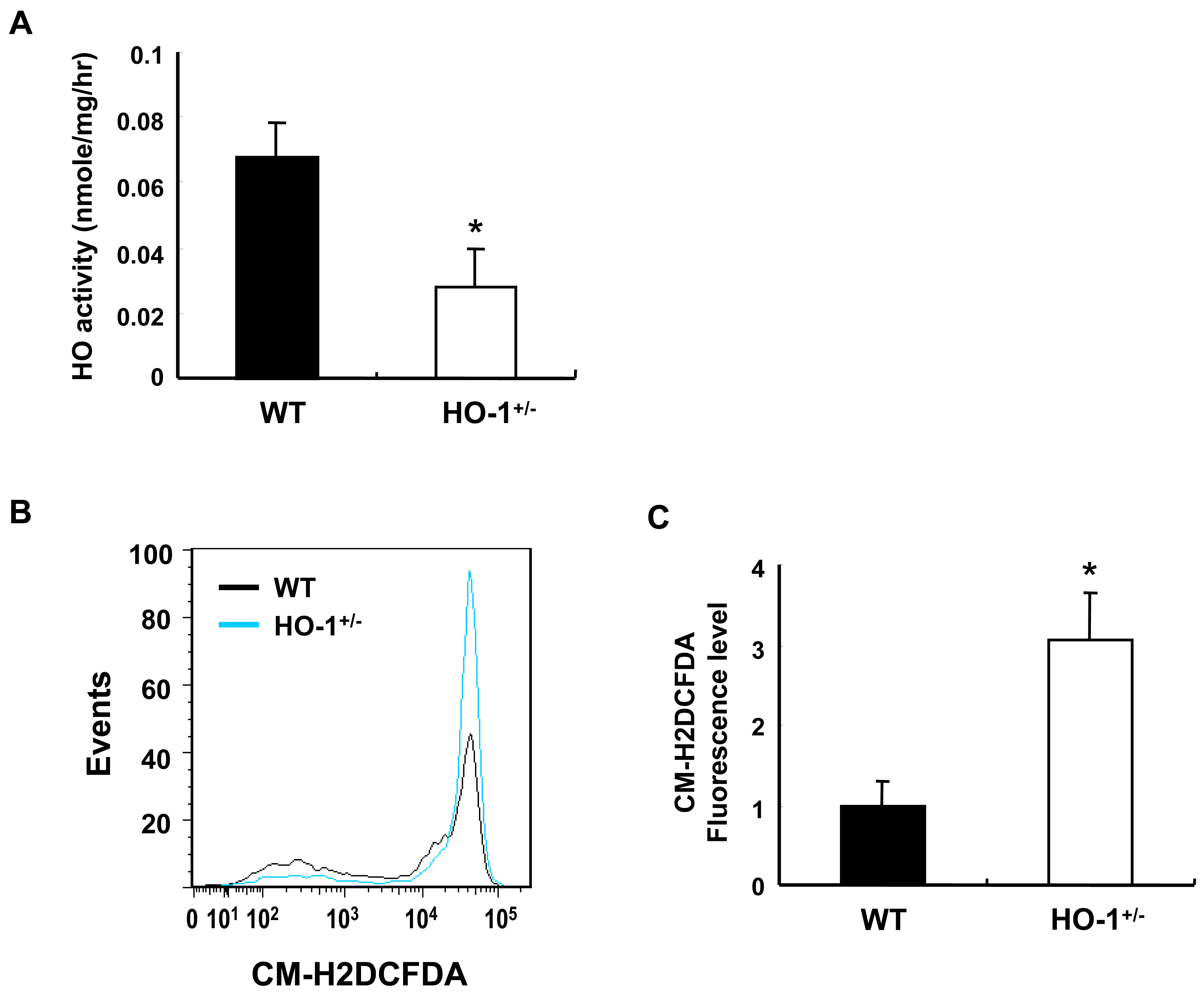

Supplement: Figure S4 — Effect of hematopoietic HO-1 haploinsufficiency on HO activity and intracellular ROS level in macrophages. Peritoneal macrophages were isolated from WT and HO-1+/− mice receiving thioglycollate injection for 4 days. A, HO activity in whole cell lysates was determined and expressed as nmoles of bilirubin produced per mg proteins per h. The number of mice in each genotype is five. *P<0.05 vs WT group. B, The levels of ROS in WT and HO-1+/− macrophages were assessed by incubation with a redox-sensitive fluorescent dye, CM-H2DCFDA, followed by flow cytometry. C, Quantitative results of CM-H2DCFDA fluorescence. The numbers of mice in WT and HO-1+/− genotypes are three and four, respectively. *P<0.05 vs WT group. (TIF) [file pone.0038626.s004.tif]
